# A Case of Successful Extraction of a Micra Leadless Pacemaker Implanted Two Years Prior Using the Axis-Guided Dual Snare Technique

**DOI:** 10.1155/cric/4729674

**Published:** 2025-05-09

**Authors:** Misun Pak, Ken Kakita, Takashi Yamasaki, Tetsuhisa Hattori

**Affiliations:** Arrhythmia Care Center, Koseikai Takeda Hospital, Kyoto, Japan

## Abstract

A 78-year-old man underwent Micra AV implantation due to complete atrioventricular block. He developed diffuse left ventricular systolic dysfunction and dyssynchrony, 2 years later, suggesting pacing-induced cardiomyopathy. Given the critical need for an upgrade to cardiac resynchronization therapy (CRT), an extraction of the Micra AV was scheduled. Initially, the Micra extraction using a solitary snare catheter was attempted, but capturing the retrieval feature on the proximal side of the device was unsuccessful due to excessive movement caused by the heartbeats. Consequently, an axis-guided dual snare technique employing two snare catheters was implemented. A triple-loop snare catheter was inserted into a steerable sheath, which was threaded through the loop of a single-loop snare catheter placed outside the sheath. The triple-loop snare successfully grasped the Micra body and stabilized its movement. Subsequently, the single-loop snare was advanced along the shaft of the triple-loop snare catheter towards the retrieval feature. The single-loop snare finally captured the retrieval feature, enabling the smooth retraction of the Micra into the sheath. Following the successful extraction of the Micra, a CRT device was implanted without complications.

## 1. Introduction

The Micra transcatheter pacing system (Micra TPS, Medtronic, Minneapolis, Minnesota) is a leadless pacemaker that offers several advantages over traditional transvenous pacemakers, including resistance to infections and avoidance of venous occlusions [1, 2]. A Micra extraction is sometimes necessary due to elevated pacing thresholds or the need to upgrade to a cardiac resynchronization therapy (CRT) device. Generally, the outcomes of Micra extractions in the chronic phase are relatively acceptable, and some successful cases of late Micra retrievals have been reported [3–5]. For a safe device removal, it is crucial to grasp the neck of the retrieval feature on the proximal side of the Micra [6, 7]. Although snaring the neck of the retrieval feature is necessary for extraction, its small structure requires precise and delicate snare manipulation. This is particularly challenging when the Micra moves significantly due to heartbeats or when the device is encapsulated by a fibrous tissue [8–11]. This case report details the successful extraction of a Micra implanted 2 years prior by the axis-guided dual snare technique, which smoothly captured the retrieval feature using two snare catheters.

## 2. Case Presentation

A 78-year-old man underwent a Micra AV implantation due to complete atrioventricular block. A Micra AV was implanted on the right ventricular (RV) mid-septum, and all electrical parameters were acceptable. However, he developed heart failure with a reduced left ventricular ejection fraction dropping from 60% before the implantation to 35% 2 years later. Transthoracic echocardiography revealed left ventricular dyssynchrony. The possibility of cardiomyopathies, including ischemic heart disease, was ruled out, and he was finally diagnosed with pacing-induced cardiomyopathy (PICM). Consequently, an upgrade to a CRT device was deemed essential, and the Micra extraction was scheduled.

The Micra extraction was performed under general anesthesia in preparation for any emergent surgeries. Initially, a Micra TPS introducer sheath (outer diameter: 27-French, Medtronic, Minneapolis, Minnesota) was inserted via the right femoral vein. To prevent back bleeding, a short sheath (outer diameter: 18-French) was introduced into the Micra sheath, followed by a steerable sheath (Agilis NXT, outer diameter: 8.5-French, Abbott, Santa Clara, California) (sheath-in-sheath technique) [12]. We initially attempted its removal using a solitary snare catheter. A triple-loop EN Snare Endovascular Snare System (Merit Medical System, South Jordan, Utah) was advanced through the steerable sheath to capture the retrieval feature on the proximal side of the Micra. However, grasping the small retrieval feature proved challenging due to the device's significant pivotal motion caused by the heartbeats, despite the triple-loop snare successfully grasping the Micra body.

**Figure 1:**
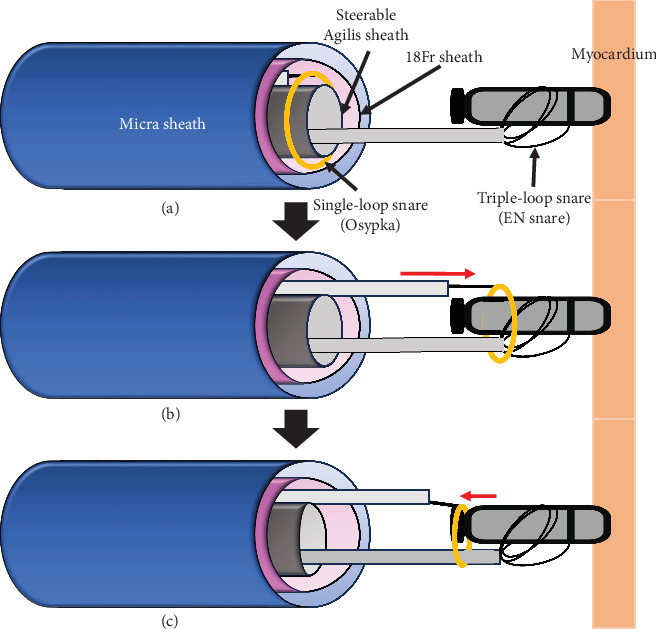
The schemas of the axis-guided dual snare technique. (a) A triple-loop EN Snare catheter was inserted into a steerable Agilis sheath, which was threaded through a loop of a single-loop Osypka snare catheter placed outside the sheath. This system was inserted together into the 18-Fr short sheath. (b) The triple-loop EN snare grasped the Micra body and stabilized its excessive movement. The single-loop Osypka snare was able to be advanced along the shaft of the triple-loop EN snare catheter towards the Micra body. (c) The single-loop Osypka snare was gradually pulled back from the body to the proximal side of the Micra while its loop was made smaller, ultimately snaring the neck of the retrieval feature.

**Figure 2:**
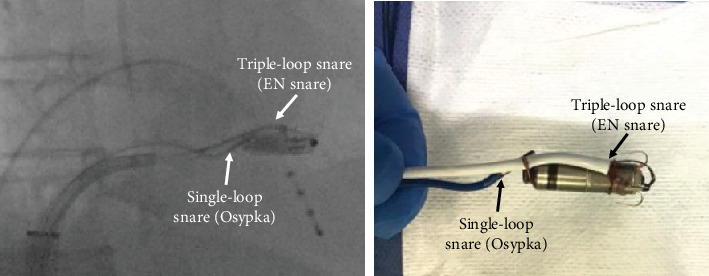
The actual images of the axis-guided dual snare technique. By holding both the Micra body and the retrieval feature with the snare catheters, the alignment of the Micra and steerable Agilis sheath could be maintained coaxially, which facilitated a smooth retrieval into the Micra sheath.

After spending over 30 min trying to capture the retrieval feature by a solitary snare catheter, a Micra extraction using two snare catheters was subsequently attempted. Initially, a triple-loop EN snare catheter was inserted into a steerable Agilis sheath, which was threaded through a loop of a single-loop snare catheter (Osypka Medical GmBH, Berlin, Germany) placed outside the sheath. This system was inserted together into the 18-Fr short sheath (Figure 1a). The triple-loop EN snare successfully grasped the Micra body and stabilized its excessive movement. Following this, the single-loop Osypka snare could be advanced along the shaft of the triple-loop EN Snare towards the Micra body (Figure 1b). The single-loop Osypka snare was gradually pulled back from the body to the proximal side of the Micra while its loop was made smaller, ultimately capturing the neck of the retrieval feature and the shaft of the EN Snare simultaneously (Figure 1c). By holding both the Micra body and retrieval feature with the two snare catheters, the alignment of the Micra and the steerable Agilis sheath could be maintained coaxially, which facilitated a smooth retrieval into the Micra sheath (axis-guided dual snare method) (Figure 2). It took only about 5 min to insert two snares into the sheath, extract the Micra, and pull back it into the sheath. After the Micra extraction, a CRT device was implanted without any complications.

## 3. Discussion

In this case, a Micra AV implanted 2 years prior was successfully removed using an axis-guided dual snare technique. This technique enabled operators to easily capture the Micra's retrieval feature by advancing one snare along the axis of another snare that was grasping the Micra's body.

**Figure 3:**
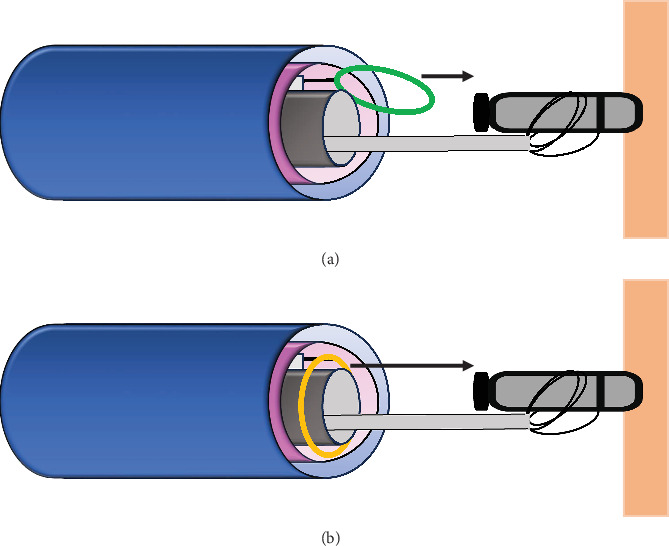
A comparison between the conventional technique and axis-guided dual snare technique. (a) Conventional technique: The Micra body is held with one snare, and another snare is inserted from outside a steerable sheath to grasp the retrieval feature. Since these snares are inserted into the sheath separately, their shafts cannot become coaxial. (b) The axis-guided dual snare technique: One snare can capture the retrieval feature more easily than the conventional technique by simply proceeding straight along the shaft of another snare catheter holding the Micra body. This technique also simplifies the device retraction into the Micra sheath by maintaining coaxial alignment between the long axis of the Micra body and the Micra sheath.

For successful Micra extractions in the chronic phase, the double-snare technique has been previously reported. This method involves firmly grasping the Micra's body with the initial snare and catching the retrieval feature with the second snare, which assists in device extraction [6]. However, this technique still requires the additional effort of capturing the small retrieval feature with the second snare. It may be hard to capture the retrieval feature from scratch, even if excessive movement of the Micra can be controlled with the initial snare (Figure 3a). On the other hand, the axis-guided dual snare technique is considered to be more effective for Micra extractions compared to the conventional approach. The key point of this technique is that the initial snare catheter is threaded through the loop of the second snare in advance. Consequently, the retrieval feature can be easily captured by simply advancing the second snare along the shaft of the initial snare holding the Micra body (“axis-guided”), eliminating the need for the burdensome manipulation of the second snare to catch the retrieval feature (Figure 3b). In this case, the solitary snare method failed to capture the retrieval feature despite significant effort. On the other hand, the axis-guided dual snare technique proved to be faster and more effective, simplifying the procedure. Since device extraction is often followed by the implantation of a new pacemaker or CRT, reducing the procedure time benefits both patients and operators. Therefore, this method is a practical and reliable first-choice approach for Micra extraction.

In our method, the Micra was removed by simultaneously capturing both the retrieval feature and the shaft of the En snare holding the Micra body with the Osypka snare catheter. Ideally, grasping only the retrieval feature is preferable in Micra retrieval. However, it was assumed that the Micra could be reliably extracted due to the strong gripping force of the Osypka snare catheter, even when both the retrieval feature and the shaft of the En snare were grasped simultaneously [13].

This case report highlights the utility of the axis-guided dual snare technique. However, its feasibility and reproducibility are uncertain, necessitating further studies with larger datasets. Additional data would help confirm the consistency and broader applicability of this technique. Furthermore, even with advancements in Micra extraction techniques, the extraction procedures are recommended to be performed in facilities equipped with cardiac surgical teams to address potential complications such as cardiac perforation or accidental dislodgments of the Micra during the extraction procedure [14, 15].

## 4. Conclusion

A Micra implanted 2 years prior was completely extracted using the axis-guided dual snare technique. This method can be considered as a potential first-choice for Micra extraction.

## Data Availability

The data that support the findings of this case report are available on request from the corresponding author. The data are not publicly available due to privacy or ethical restrictions.
